# Quality of Life in Patients with Atrial Fibrillation Undergoing Pulmonary Vein Isolation: Short-Term Follow-Up Study

**DOI:** 10.3390/medicina60101594

**Published:** 2024-09-28

**Authors:** Matiss Linde, Kristine Jubele, Kaspars Kupics, Anastasija Nikitina, Andrejs Erglis

**Affiliations:** 1 Pauls Stradins Clinical University Hospital, LV-1002 Riga, Latvia; 2 Faculty of Medicine, Riga Stradins University, LV-1007 Riga, Latvia; 3 Faculty of Medicine and Life Sciences, University of Latvia, LV-1004 Riga, Latvia

**Keywords:** atrial fibrillation, pulmonary vein isolation, quality of life

## Abstract

*Background and Objectives:* Atrial fibrillation (AF) significantly impacts the quality of life (QoL) of affected individuals. Pulmonary vein isolation (PVI) has emerged as a therapeutic approach to manage AF and improve QoL. This study aimed to assess the QoL in patients with AF undergoing PVI. *Methods and Materials:* A total of 97 AF patients undergoing PVI (radiofrequency 52.6% (n = 51) and cryoablation 47.4% (n = 46)) at Pauls Stradins Clinical University Hospital were included in this study. QoL was measured using the 36-Item Short-Form Survey (SF-36) before PVI and during a follow-up period of 5.98 ± 1.97 months. *Results:* This study consisted of 60.8% (n = 59) males, with a mean age of 60.06 ± 11.61 years. A total of 67.0% (n = 65) of patients had paroxysmal AF, and 33.0% (n = 32) had persistent AF. The SF-36 questionnaire revealed major improvements across multiple QoL domains post-PVI, reaching a statistical significance of *p* < 0.01. Patient factors, such as female gender ([estimate 21.26, 95% CI (7.18, 35.35)], *p* < 0.01), persistent AF ([estimate 15.49, 95% CI (2.83, 28.15)], *p* = 0.02), and restored sinus rhythm ([estimate 14.35, 95% CI (1.65, 27.06)], *p* = 0.03), were associated with significantly improved QoL. *Conclusions:* PVI in patients with AF positively influences various dimensions of QoL, as evidenced by significant improvement across multiple SF-36 domains. These findings emphasize worsened QoL in patients with AF and the potential benefits of PVI enhancing the overall wellbeing of individuals with AF.

## 1. Introduction

Atrial fibrillation (AF), the most prevalent form of arrhythmia, often presents with symptoms such as dyspnea, chest pain, fatigue, dizziness, and in some cases, syncope. However, its manifestation can also be asymptomatic [1]. Notably, AF is associated with life-threatening complications, including stroke, and can significantly impair quality of life (QoL). Recent data also demonstrated an increase in the burden and cost of health care related to AF, especially in individuals with paroxysmal AF [2]. Fortunately, multiple treatment options exist to ameliorate QoL by restoring sinus rhythm. Among these, pulmonary vein isolation (PVI) has gained prominence alongside conventional pharmacological treatment [3,4]. So far, PVI is most commonly performed using either radiofrequency or cryoballoon ablation techniques [5].

Numerous studies have documented improvements in QoL following ablation therapy [6,7,8]. To assess QoL, various questionnaires are available. In our study, we employed the widely recognised and accessible 36-Item Short-Form Survey Instrument (SF-36) [9] to evaluate QoL in patients with AF undergoing PVI. This choice reflects our commitment to utilising a comprehensive and reliable measure for the assessment of QoL post-treatment.

## 2. Materials and Methods

### 2.1. Study Design

This was a single-centre prospective cohort study performed at the Latvian Centre of Cardiology, Pauls Stradins Clinical University Hospital. Patients with paroxysmal or persistent AF who were scheduled for PVI from July 2022 to July 2023 were offered to participate in this study. The SF-36 questionnaire was used to assess QoL before and after PVI, either with radiofrequency ablation or cryoablation.

The SF-36 consists of 36 questions that cover eight health domains, and the responses are used to generate scores for each domain. These eight health domains are grouped into two main physical and mental components. Some questions were rephrased so that the questionnaire’s domain and subdomain scores were positive, ranging from 0 to 100, without losing the context of the questions.

Patients were admitted to our centre one day prior to PVI. On the same day, the SF-36 questionnaire was given to patients who gave written consent to participate in this study. Considering the blanking period, that is defined as post-procedural phase after PVI during which transient atrial arrhythmias are common and not considered indicative of long-term procedural failure, follow-ups began 3 months after the procedure. During this period, all patients were prescribed antiarrhythmic drug therapy for at least 3 months. Patients scheduled a follow-up appointment with an arrhythmologist. Those patients who did not make an appointment in 3 months were contacted via telephone and offered an appointment. The questionnaire was completed before consultation with an arrhythmologist. Patients who were not able to come in for an appointment answered the questionnaire via telephone. This study was approved by the local ethics committee and patients gave written consent for their participation. Participation in this study was offered to all patients admitted for PVI.

### 2.2. Ablation Procedure

In all patients, the puncture of the intra-atrial septum was performed under transoesophageal echocardiography and X-ray control. In the radiofrequency group, 3D mapping of left atrium was performed using a pentaray catheter and CARTO 3 system (Biosense Webster, Inc., Diamond Bar, CA, USA). Circular linear lesions were created around the ostia of the pulmonary veins. Isolation of veins was confirmed using a pentaray catheter and remapping the left atrium. In the cryotherapy group, a cryoballoon was inserted in the left atrium, and the pulmonary vein occlusion was performed under X-ray control using contrast injections. The isolation of pulmonary veins was confirmed using a circular catheter. The isolation of all veins was achieved during the procedure in both groups.

### 2.3. Statistical Analysis

Categorical data from the SF-36 questionnaire were converted into continuous data, according to SF-36 scoring instructions [10]. Other categorical data were represented as frequencies. Normality was assessed using both visual inspection of a normal probability plot and the Shapiro–Wilk test. Subsequently, non-parametric Wilcoxon signed-rank test for non-normal data and the parametric paired-sample t-test for normal data were performed. To further understand PVI efficacy on QoL, we conducted subgroup analysis by employing previously mentioned tests. We were cautious interpreting the results of the subgroups that showed no significant difference, because a post hoc power analysis demonstrated a statistical power of <60%. Linear regression analysis was performed to identify factors significantly associated with improvement to QoL. The QoL improvement, measured as the difference in the QoL scores from baseline to follow-up, served as the dependent variable. Prior to conducting the regression analysis, diagnostic tests were performed to ensure that the model’s assumptions were met, including linearity, homoscedasticity, the independence of residuals, and normal distribution of errors. All statistical analyses were conducted using IBM SPSS Statistics 22.0, and a significance level of *p* < 0.01 was chosen to indicate statistical significance.

## 3. Results

### 3.1. Patient Characteristics

A total of 60.8% (n = 59) of males and 39.2% (n = 38) of females were included in this study with either 67.0% (n = 65) paroxysmal or 33.0% (n = 32) persistent AF. A description of the study population can be seen in Table 1. The mean age of the population was 60.06 ± 11.61 years, and most patients did not have multiple comorbidities; for example, 24.7% (n = 24) of patients had chronic heart failure, 10.3% (n = 10) had diabetes mellitus, and only 3.1% (n = 3) had chronic kidney disease. However, more than half of the patients had primary arterial hypertension, which is one of the leading causes of atrial fibrillation. A total of 52.6% (n = 51) of patients underwent radiofrequency ablation and 47.4% (n = 46) underwent cryoablation.

### 3.2. Quality of Life Assessment

QoL was assessed in 8 domains in addition to examining health change perception at baseline and collecting mean 6-month follow-up data: physical functioning (63.35 ± 23.94 vs. 82.98 ± 21.03, *p* < 0.01), role limitations due to physical health (36.05 ± 40.07 vs. 75.29 ± 38.31, *p* < 0.01), role limitations due to emotional problems (53.26 ± 43.74 vs. 82.13 ± 32.65, *p* < 0.01), energy/fatigue (52.88 ± 17.99 vs. 70.10 ± 13.10, *p* < 0.01), emotional wellbeing (63.21 ± 18.16 vs. 54.43 ± 20.64, *p* < 0.01), social functioning (73.84 ± 22.32 vs. 84.89 ± 12.15, *p* < 0.01), pain (76.65 ± 21.08 vs. 84.45 ± 14.06, *p* < 0.01), general health (48.91 ± 17.14 vs. 60.30 ± 15.52, *p* < 0.01), and health change (44.32 ± 21.50 vs. 76.28 ± 18.53, *p* < 0.01). There was a statistically significant increase in QoL in these domains. Analysing the answers in detail revealed clues for a persisting psychological burden; for example, there was not a statistically significant difference in subdomains of feeling uplifted and positive, or agitated and restless. Additionally, patients perceived that their immune system did not change, implying a simplistic view of their own health, reducing their awareness of modifiable risk factors. Table 2 represents mean scores with standard deviations at baseline and follow-up with respect to each SF-36 domain and subdomain.

**Figure 1:**
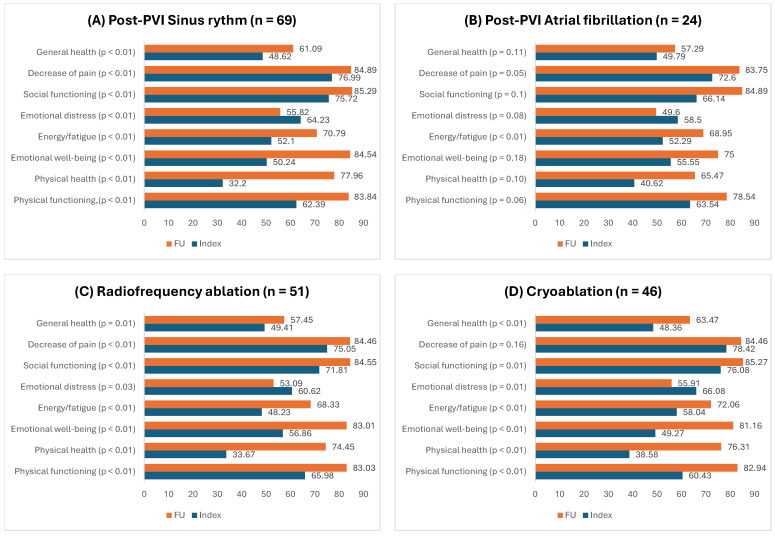
Changes in quality of life after pulmonary vein isolation in sub-populations, represented with mean score values.

The documented sinus rhythm on follow-up was restored and maintained in 74.2% (n = 69) of patients. A total of 4 patients who were followed up via telephone did not have a documented rhythm, but still answered the questionnaire. There were statistically significant changes to QoL between patients who underwent radiofrequency ablation and cryoablation, showing that both methods were effective at improving QoL. There was a notable difference between patients who had documented sinus rhythms and those who had AF after PVI. Patients who had documented AF after PVI statistically showed significant improvement in social functioning and energy/fatigue domains, but patients who had a sinus rhythm statistically showed significant improvement in all QoL domains. QoL changes in these sub-populations are represented in Figure 1.

**Figure 2:**
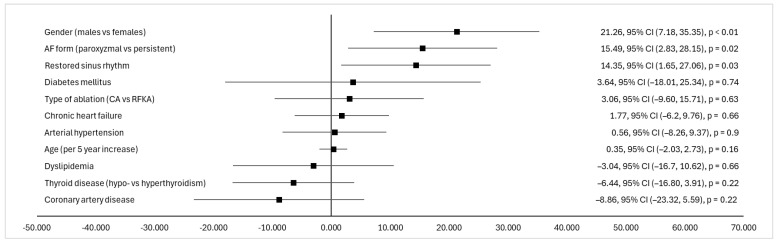
Factors associated with QoL represented with linear regression estimated values. AF—atrial fibrillation; CA—cryoablation; RFKA—radiofrequency ablation.

There was a significant QoL difference between males and females following the intervention ([estimate 21.26, 95% CI (7.18, 35.35)], *p* < 0.01). Females experienced a substantially higher improvement in QoL than males. This highlights the importance of considering gender in the management and treatment outcomes of atrial fibrillation. The form of atrial fibrillation significantly impacts QoL ([estimate 15.49, 95% CI (2.83, 28.15)], *p* = 0.02), indicating that paroxysmal AF is associated with a worse QoL compared with persistent AF. This could suggest that the treatment that is applied as AF progresses might lead to perceived improvements in QoL, possibly due to better symptom management or adaptation over time. Patients who had their sinus rhythm restored and maintained after PVI showed a statistically significant improvement in the QoL difference ([estimate 14.35, 95% CI (1.65, 27.06), *p* = 0.03) compared to those who did not maintain sinus rhythm. Other factors were not statistically significant, suggesting that they do not have a significant independent effect on QoL. Factors associated with QoL are represented in Figure 2.

### 3.3. Complications and Medical Therapy

A total of 5.2% (n = 5) of patients had periprocedural complications: 1 had femoral arteriovenous fistula following the suturing of the artery; 1 had iatrogenic cardiac tamponade following the pericardiocentesis; 1 had right profunda femoris artery extravasation managed without surgical intervention; and 2 had phrenic nerve injury. Post procedure, all patients continued anticoagulant therapy. Mainly, direct oral anticoagulants were prescribed. Only 7.2% (n = 7) of patients were prescribed Warfarin. As an important aspect of maintaining sinus rhythm, 92.8% (n = 90) of patients were prescribed antiarrhythmics.

## 4. Discussion

This study aimed to assess QoL by using the SF-36 questionnaire in patients with AF undergoing PVI. The main finding was a statistically significant improvement in the QoL across the assessed domains. Improvement was noted in areas such as physical functioning, role limitations due to physical health, emotional wellbeing, energy/fatigue, emotional distress, social functioning, pain, general health, and perceived health change. Despite overall improvements to QoL, there was the indication of a persisting psychological burden. For instance, no significant difference was observed in subdomains related to feeling uplifted or agitated and restless after PVI. In females, persistent AF and restored sinus rhythm were significantly associated with better QoL, while age and comorbidities did not show a significant effect on QoL.

SF-36 has been referenced as a validated tool to be used to assess QoL in AF patients [9] and many studies have used it to evaluate the effect on QoL after PVI [11,12,13]. It could be argued that the drawback of SF-36 is that it assesses overall health and functioning, instead of focusing on symptoms unique to AF. Consequently, the results can be affected by factors such as patient demographics and comorbidities, particularly in elderly AF patients. Interestingly, one study showed that AF patients who underwent PVI improved their SF-36 subscale scores to levels comparable to those of an age-matched healthy control population, and concluded that demographic or clinical variables, as well as baseline QoL scores, were not predictive of the response to PVI [11]. There are other questionnaires available, such as AFEQT [14] and MAFSI [15], that evaluate specific AF symptoms, but SF-36 provides a comprehensive overview of general health, including physical, mental, and social functioning, thus giving a holistic view of a patient’s QoL.

According to the guidelines for the management of AF, catheter ablation is recommended to improve QoL in AF patients [16]. There are several studies that show greater improvement in QoL due to catheter ablation compared to medical therapy. For example, in the EAST-AFNET 4 study, patients were divided into early rhythm control or usual care groups. The primary outcome of cardiovascular events was more prevalent in the group receiving the usual care for AF. However, at 2 years, QoL did not differ between the two groups [3]. Additionally, in the CABANA trial, catheter ablation resulted in significantly better QoL outcomes at 12 months compared to medical therapy, and was more effective in reducing the severity and frequency of AF symptoms compared to medical therapy [4]. STOP-AF [17] and EARLY-AF [18] trials demonstrated that cryoablation as a first-line therapy is more effective in restoring sinus rhythm than medical therapy. It could be argued that catheter ablation should be used as a first-line therapy option for AF patients if antiarrhythmics have not been administered. This strategy would benefit patients, because the burden of medication is associated with lower adherence and negative psychological aspects, especially in elderly patients [19]. Additionally, there is evidence that PVI can reduce psychological factors such as heart-focused anxiety, general anxiety, and depressive symptoms [20].

In addition to well-established ablation methods, the latest technology of pulse field ablation comes with promising results. The EU-PORIA study demonstrated a similar safety profile to radiofrequency ablation and cryoablation, but with fewer major complications [21]. Moreover, the PULSED AF study showed that PFA led to significant improvements in patient quality of life, indicating its potential as a safer and efficient alternative to current methods with a low major complication rate [22].

There are promising methods with which to reduce the use of antiarrhythmic medication and still restore sinus rhythm and improve QoL; but in our study, patients used antiarrhythmics prior to PVI, and 92.8% of patients continued using them for at least 3 months post-PVI. This conventional approach could be changed, as there is evidence from the POWDER-AF2 trial [23] that beyond the blanking period, there is mostly no difference in continuing or discontinuing antiarrhythmic therapy to maintain sinus rhythm.

Despite the efficacy of PVI in treating AF, comorbidities should be considered as comparable. De novo AF is rare and mostly occurs from underlying disease [24]. This could have greater importance to QoL, especially for polymorbid patients. Other factors than comorbidities play important roles on QoL; for example, there is evidence that for females, and patients of a younger age, new-onset AF is associated with a lower QoL [25].

Compared to the above-mentioned studies [3,4,17,18], our study highlights and confirms similar improvements in QoL, especially those that are determined by factors such as female gender and patients with persistent AF. The restoration of sinus rhythm was crucial for comprehensive QoL enhancement. Interestingly, comorbidities and age did not show a significant effect on QoL. We also revealed a persistent psychological burden, such as restlessness, despite overall QoL improvements. Additionally, we compared the effectiveness of radiofrequency ablation and cryoablation on QoL, finding both beneficial.

Even though QoL showed significant improvement, there might be some study limitations, such as, small sample size, short follow-up time, and reliance on self-reported measures, which can introduce response bias and may not accurately reflect true QoL.

## 5. Conclusions

This study demonstrated that PVI significantly improves the overall QoL of patients with AF across various health domains, though there is evidence of persisting psychological factors post-treatment. While PVI is effective, we would like to emphasise the importance of considering patient factors, comorbidities, and the impact of ongoing antiarrhythmic medication, highlighting a need for personalised treatment approaches in AF management.

## Figures and Tables

**Table 1:** Characteristics of the population.

Characteristics	n = 97 (%)
Gender	
Male	59 (60.80)
Female	38 (39.20)
Age	60.06 ± 11.61
Follow up duration, months	5.98 ± 1.97
Atrial fibrillation	
Paroxysmal	65 (67.00)
Persistent	32 (33.00)
Type of ablation	
Radiofrequency	51 (52.60)
Cryoablation	46 (47.40)
Coronary artery disease	18 (18.60)
History of myocardial infarction	2 (2.10)
Chronic heart failure	24 (24.74)
NYHA I	6 (6.30)
NYHA II	17 (17.70)
NYHA III	1 (1.00)
Primary arterial hypertension	56 (57.73)
Grade 1	18 (18.60)
Grade 2	36 (37.10)
Grade 3	2 (2.10)
Chronic kidney disease	3 (3.1)
Diabetes mellitus	10 (10.3)
Dyslipidemia	25 (25.8)
Hypothyroidism	10 (10.4)
Hyperthyroidism	1 (1.0)

**Table 2:** Quality of life in patients with atrial fibrillation after PVI, assessed with SF-36 questionnaire.

Item		Index (n = 97)		Follow-Up (n = 97)		*p*-Value
		Mean ± SD	Median (Q1–Q3)	Mean ± SD	Median (Q1–Q3)	
**Physical functioning**		**63.35** **± 23.94**	**65 (45–85)**	**82.98** **± 21.03**	**90 (72.5–100)**	**<0.01**
	Vigorous activities, such as running, lifting heavy objects, participating in strenuous sports	25.77 ± 37.57	0 (0–50)	58.76 ± 42.08	50 (0–100)	<0.01
	Moderate activities, such as moving a table, pushing a vacuum cleaner, bowling, or playing golf	65.97 ± 33.49	50 (50–100)	80.92 ± 26.46	100 (50–100)	<0.01
	Lifting or carrying groceries	62.37 ± 36.10	50 (50–100)	82.98 ± 30.52	100 (50–100)	<0.01
	Climbing several flights of stairs	43.81 ± 35.54	50 (0–50)	76.80 ± 34.65	100 (50–100)	<0.01
	Climbing one flight of stairs	79.90 ± 30.33	100 (50–100)	92.78 ± 17.66	100 (100–100)	<0.01
	Bending, kneeling, or stooping	61.34 ± 37.85	50 (50–100)	83.50 ± 26.73	100 (50–100)	<0.01
	Walking more than a mile	67.71 ± 38.37	100 (50–100)	85.05 ± 27.16	100 (50–100)	<0.01
	Walking several blocks	61.45 ± 38.02	50 (50–100)	81.95 ± 31.61	100 (50–100)	<0.01
	Walking one block	77.60 ± 31.51	100 (50–100)	89.69 ± 24.93	100 (100–100)	<0.01
	Bathing or dressing yourself	88.54 ± 22.33	100 (50–100)	97.42 ± 11.11	100 (100–100)	<0.01
**Role enhancements due to physical health**		**36.05** **± 40.07**	**25 (0–75)**	**75.29** **± 38.31**	**100 (50–100)**	**<0.01**
	Increased the amount of time you spent on work or other activities.	46.31 ± 50.12	0 (0–100)	79.76 ± 40.41	100 (100–100)	<0.01
	Accomplished more than you would like.	36.45 ± 48.38	0 (0–100)	75.25 ± 43.37	100 (100–100)	<0.01
	Expanded the range of work or other activities.	27.83 ± 45.05	0 (0–100)	76.28 ± 42.75	100 (100–100)	<0.01
	Experienced ease in performing the work or other activities.	33.33 ± 47.38	0 (0–100)	77.31 ± 42.09	100 (100–100)	<0.01
**Role enhancements due to emotional well-being**		**53.26 ± 43.74**	**66.6 (0–100)**	**82.13** **± 32.65**	**100 (66.6–100)**	**<0.01**
	Increased the amount of time you spent on work or other activities.	52.57 ± 50.19	100 (0–100)	83.50 ± 37.30	100 (100–100)	<0.01
	Accomplished more than you would like.	47.42 ± 50.19	0 (0–100)	76.28 ± 42.75	100 (100–100)	<0.01
	Did work or other activities with extra care and attention.	59.79 ± 49.28	100 (0–100)	86.59 ± 34.24	100 (100–100)	<0.01
**Energy/fatigue**		**52.88 ± 17.99**	**50 (40–70)**	**70.10** **± 13.10**	**75 (62.5–75)**	**<0.01**
	Did you feel full of pep?	47.42 ± 23.15	40 (20–60)	81.03 ± 22.05	80 (60–100)	<0.01
	Did you have a lot of energy?	48.86 ± 23.97	40 (40–60)	66.59 ± 17.49	80 (60–80)	<0.01
	Did you feel energized and refreshed?	63.09 ± 22.42	60 (40–80)	83.91 ± 20.13	100 (80–100)	<0.01
	Did you feel alert and full of energy?	52.16 ± 19.26	40 (40–60)	48.86 ± 26.29	40 (20–60)	0.34
**Emotional distress**		**63.21 ± 18.16**	**64 (52–76)**	**54.43** **± 20.64**	**56 (36–68)**	**<0.01**
	Have you been a very calm and composed person?	59.58 ± 22.16	60 (40–80)	51.13 ± 19.78	40 (40–60)	<0.01
	Have you felt consistently uplifted and positive?	74.63 ± 23.18	80 (60–100)	74.84 ± 16.14	80 (60–80)	0.83
	Have you felt agitated and restless?	58.76 ± 24.80	60 (40–80)	56.28 ± 28.03	60 (50–80)	0.67
	Have you felt optimistic and cheerful?	68.86 ± 23.79	60 (60–80)	55.67 ± 30.71	60 (20–80)	<0.01
	Have you been an unhappy person?	54.22 ± 19.99	60 (40–60)	34.22 ± 34.63	20 (0–60)	<0.01
**Social functioning**		**73.84 ± 22.32**	**75 (62.5–87.5)**	**84.89** **± 12.15**	**87.5 (75–87.5)**	**<0.01**
	During the past 4 weeks, to what extent has your physical health or emotional well-being enhanced your normal social activities with family, friends, neighbors, or groups?	67.01 ± 25.39	75 (50–75)	76.03 ± 15.69	75 (75–75)	<0.01
	During the past 4 weeks, how much of the time has your physical health or emotional well-being allowed for or contributed positively to your social activities (like visiting with friends, relatives, etc.)?	80.67 ± 24.34	100 (50–100)	93.75 ± 12.56	100 (100–100)	<0.01
**Pain**		**76.65 ± 21.08**	**77.5 (55–100)**	**84.45** **± 14.06**	**90 (77.5–90)**	**<0.01**
	No bodily pain experienced during the past 4 weeks	78.55 ± 18.76	80 (60–100)	84.12 ± 14.12	80 (80–100)	0.01
	Pain did not interfere with normal work at all during the past 4 weeks	74.74 ± 25.38	75 (50–100)	84.79 ± 19.28	100 (75–100)	<0.01
**General health**		**48.91 ± 17.14**	**45 (35–60)**	**60.30** **± 15.52**	**55 (55–75)**	**<0.01**
	How would you assess your general health?	31.44 ± 19.18	25 (25–50)	62.62 ± 23.96	75 (50–75)	<0.01
	I seem to have a stronger immune system than other people?	66.75 ± 25.44	75 (50–100)	64.17 ± 27.69	50 (50–100)	0.61
	I am as healthy as anybody I know	47.42 ± 27.83	50 (25–75)	55.67 ± 26.64	75 (50–75)	<0.01
	I expect my health to improve or stay the same?	64.69 ± 27.18	50 (50–100)	76.28 ± 19.88	75 (75–100)	<0.01
	My health is excellent	34.27 ± 30.47	25 (0–50)	42.78 ± 35.34	50 (0–75)	0.06
**Health change**		**44.32** **± 21.50**	**50 (25–50)**	**76.28** **± 18.53**	**75 (75–100)**	**<0.01**

## Data Availability

The original contributions presented in the study are included in the article, further inquiries can be directed to the corresponding author.

## References

[1] Guerra F., Brambatti M., Nieuwlaat R., Marcucci M., Dudink E., Crijns H.J.G.M., Matassini M.V., Capucci A. (2017). Symptomatic atrial fibrillation and risk of cardiovascular events: Data from the Euro Heart Survey. Europace.

[2] Peigh G., Zhou J., Rosemas S.C., Roberts A.I., Longacre C., Nayak T., Schwab G., Soderlund D., Passman R.S. (2024). Impact of Atrial Fibrillation Burden on Health Care Costs and Utilization. JACC Clin. Electrophysiol..

[3] Kirchhof P., Camm A.J., Goette A., Brandes A., Eckardt L., Elvan A., Fetsch T., van Gelder I.C., Haase D., Haegeli L.M. (2020). EAST-AFNET 4 Trial Investigators. Early Rhythm-Control Therapy in Patients with Atrial Fibrillation. N. Engl. J. Med..

[4] Mark D.B., Anstrom K.J., Sheng S., Piccini J.P., Baloch K.N., Monahan K.H., Daniels M.R., Bahnson T.D., Poole J.E., Rosenberg Y. (2019). CABANA Investigators. Effect of Catheter Ablation vs Medical Therapy on Quality of Life among Patients with Atrial Fibrillation: The CABANA Randomized Clinical Trial. JAMA.

[5] Calkins H., Hindricks G., Cappato R., Kim Y.H., Saad E.B., Aguinaga L., Akar J.G., Badhwar V., Brugada J., Camm J. (2017). 2017 HRS/EHRA/ECAS/APHRS/SOLAECE expert consensus statement on catheter and surgical ablation of atrial fibrillation. Heart Rhythm.

[6] Mohanty S., Mohanty P., Di Biase L., Bai R., Pump A., Santangeli P., Burkhardt D., Gallinghouse J.G., Horton R., Sanchez J.E. (2012). Impact of metabolic syndrome on procedural outcomes in patients with atrial fibrillation undergoing catheter ablation. J. Am. Coll. Cardiol..

[7] Andrade J.G., Macle L., Verma A., Deyell M.W., Champagne J., Dubuc M., Leong-Sit P., Novak P., Roux J.F., Sapp J. (2020). CIRCA-DOSE Study Investigators. Quality of Life and Health Care Utilization in the CIRCA-DOSE Study. JACC Clin. Electrophysiol..

[8] Biviano A.B., Hunter T.D., Dandamudi G., Fishel R.S., Gidney B., Herweg B., Oza S.R., Patel A.M., Wang H., Pollak S.J. (2017). Healthcare Utilization and Quality of Life Improvement after Ablation for Paroxysmal AF in Younger and Older Patients. Pacing Clin. Electrophysiol..

[9] Aliot E., Botto G.L., Crijns H.J., Kirchhof P. (2014). Quality of life in patients with atrial fibrillation: How to assess it and how to improve it. Europace.

[10] 36-Item Short Form Survey (SF-36) Scoring Instructions. https://www.rand.org/health-care/surveys_tools/mos/36-item-short-form/scoring.html.

[11] Pürerfellner H., Martinek M., Aichinger J., Nesser H.J., Kempen K., Janssen J.P. (2004). Quality of life restored to normal in patients with atrial fibrillation after pulmonary vein ostial isolation. Am. Heart J..

[12] Xu Y., Sharma D., Du F., Li G., Xu G. (2012). Comparison of circumferential pulmonary vein isolation and antiarrhythmic drug therapy in patients with atrial fibrillation. Cardiol. Ther..

[13] Woźniak-Skowerska I.M., Skowerski M.J., Hoffmann A., Nowak S., Faryan M., Kolasa J., Skowerski T., Szydło K., Wnuk-Wojnar A.M., Mizia-Stec K. (2016). Quality of life in patients with paroxysmal atrial fibrillation after circumferential pulmonary vein ablation. Pol. Heart J. (Kardiol. Pol.).

[14] Spertus J., Dorian P., Bubien R., Lewis S., Godejohn D., Reynolds M.R., Lakkireddy D.R., Wimmer A.P., Bhandari A., Burk C. (2011). Development and validation of the Atrial Fibrillation Effect on QualiTy-of-Life (AFEQT) Questionnaire in patients with atrial fibrillation. Circ. Arrhythmia Electrophysiol..

[15] Wokhlu A., Monahan K.H., Hodge D.O., Asirvatham S.J., Friedman P.A., Munger T.M., Bradley D.J., Bluhm C.M., Haroldson J.M., Packer D.L. (2010). Long-term quality of life after ablation of atrial fibrillation the impact of recurrence, symptom relief, and placebo effect. J. Am. Coll. Cardiol..

[16] Hindricks G., Potpara T., Dagres N., Arbelo E., Bax J.J., Blomström-Lundqvist C., Boriani G., Castella M., Dan G.A., Dilaveris P.E. (2021). ESC Scientific Document Group. 2020 ESC Guidelines for the diagnosis and management of atrial fibrillation developed in collaboration with the European Association for Cardio-Thoracic Surgery (EACTS): The Task Force for the diagnosis and management of atrial fibrillation of the European Society of Cardiology (ESC) Developed with the special contribution of the European Heart Rhythm Association (EHRA) of the ESC. Eur. Heart J..

[17] Wazni O.M., Dandamudi G., Sood N., Hoyt R., Tyler J., Durrani S., Niebauer M., Makati K., Halperin B., Gauri A. (2021). STOP AF First Trial Investigators. Cryoballoon Ablation as Initial Therapy for Atrial Fibrillation. N. Engl. J. Med..

[18] Andrade J.G., Wells G.A., Deyell M.W., Bennett M., Essebag V., Champagne J., Roux J.F., Yung D., Skanes A., Khaykin Y. (2021). EARLY-AF Investigators. Cryoablation or Drug Therapy for Initial Treatment of Atrial Fibrillation. N. Engl. J. Med..

[19] Yang C., Zhu S., Hui Z., Mo Y. (2023). Psychosocial factors associated with medication burden among community-dwelling older people with multimorbidity. BMC Geriatr..

[20] Pavlicek V., Wedegärtner S.M., Millenaar D., Wintrich J., Böhm M., Kindermann I., Ukena C. (2022). Heart-Focused Anxiety, General Anxiety, Depression and Health-Related Quality of Life in Patients with Atrial Fibrillation Undergoing Pulmonary Vein Isolation. J. Clin. Med..

[21] Schmidt B., Bordignon S., Neven K., Reichlin T., Blaauw Y., Hansen J., Adelino R., Ouss A., Füting A., Roten L. (2023). EUropean real-world outcomes with Pulsed field ablatiOn in patients with symptomatic atRIAl fibrillation: Lessons from the multi-centre EU-PORIA registry. Europace.

[22] Verma A., Haines D.E., Boersma L.V., Sood N., Natale A., Marchlinski F.E., Calkins H., Sanders P., Packer D.L., Kuck K.H. (2023). PULSED AF Investigators. Pulsed Field Ablation for the Treatment of Atrial Fibrillation: PULSED AF Pivotal Trial. Circulation.

[23] Demolder A., O’Neill L., El Haddad M., Scherr D., Vijgen J., Wolf M., Berte B., Bisbal F., Johannessen A., Rivero-Ayerza M. (2023). No Effect of Continued Antiarrhythmic Drug Treatment on Top of Optimized Pulmonary Vein Isolation in Patients with Persistent Atrial Fibrillation: Results From the POWDER-AF2 Trial. Circ. Arrhythmia Electrophysiol..

[24] Kozlowski D., Budrejko S., Lip G.Y., Rysz J., Mikhailidis D.P., Raczak G., Banach M. (2010). Lone atrial fibrillation: What do we know?. Heart.

[25] Randolph T.C., Simon D.N., Thomas L., Allen L.A., Fonarow G.C., Gersh B.J., Kowey P.R., Reiffel J.A., Naccarelli G.V., Chan P.S. (2016). ORBIT AF Investigators and Patients. Patient factors associated with quality of life in atrial fibrillation. Am. Heart J..

